# Parallels between Pathogens and Gluten Peptides in Celiac Sprue

**DOI:** 10.1371/journal.ppat.0040034

**Published:** 2008-02-29

**Authors:** Michael T Bethune, Chaitan Khosla

**Affiliations:** University of British Columbia, Canada

## Abstract

Pathogens are exogenous agents capable of causing disease in susceptible organisms. In celiac sprue, a disease triggered by partially hydrolyzed gluten peptides in the small intestine, the offending immunotoxins cannot replicate, but otherwise have many hallmarks of classical pathogens. First, dietary gluten and its peptide metabolites are ubiquitous components of the modern diet, yet only a small, genetically susceptible fraction of the human population contracts celiac sprue. Second, immunotoxic gluten peptides have certain unusual structural features that allow them to survive the harsh proteolytic conditions of the gastrointestinal tract and thereby interact extensively with the mucosal lining of the small intestine. Third, they invade across epithelial barriers intact to access the underlying gut-associated lymphoid tissue. Fourth, they possess recognition sequences for selective modification by an endogenous enzyme, transglutaminase 2, allowing for *in situ* activation to a more immunotoxic form via host subversion. Fifth, they precipitate a T cell–mediated immune reaction comprising both innate and adaptive responses that causes chronic inflammation of the small intestine. Sixth, complete elimination of immunotoxic gluten peptides from the celiac diet results in remission, whereas reintroduction of gluten in the diet causes relapse. Therefore, in analogy with antibiotics, orally administered proteases that reduce the host's exposure to the immunotoxin by accelerating gluten peptide destruction have considerable therapeutic potential. Last but not least, notwithstanding the power of *in vitro* methods to reconstitute the essence of the immune response to gluten in a celiac patient, animal models for the disease, while elusive, are likely to yield fundamentally new systems-level insights.

## The Expanding Concept of Pathogens

The capacity for exogenous agents to cause disease in susceptible organisms has been widely recognized since the germ theory of disease gained acceptance in the late nineteenth century. In the roughly 120 years since then, over 1,400 such disease-causing agents, termed pathogens, have been identified [1] and their respective roles in disease pathology elucidated to varying degrees.

Nonetheless, there remains considerable difficulty in defining what exactly constitutes a pathogen even today, and the definition of this term has necessarily expanded with our understanding of disease etiology [2,3]. A majority of literature definitions for the term pathogen take their cue from Koch's postulates and focus on disease-causing microorganisms, predominantly bacteria [4–6]. However, non-living infectious agents such as viruses and prions can cause disease as well, and Koch's postulates have been periodically adapted to account for such new classes of pathogens [7,8]. Prions are a particularly notable example of this conceptual expansion, being merely misfolded proteins that replicate by catalyzing the misfolding and aggregation of properly folded host prion proteins in a templated fashion [9]. Discovered only 25 years ago, these pathogenic proteins are responsible for a growing number of devastating neurodegenerative diseases [10].

Even as new pathogens capable of causing human disease are uncovered, evidence is emerging that several diseases not previously considered to have an infectious etiology may involve pathogens. Among these are hepatocellular carcinoma and type II diabetes (hepatitis C virus [11,12]), Crohn disease (Mycobacterium avium [13]), peptic ulcers and gastric carcinoma (Helicobacter pylori [14,15]), cervical carcinoma (human papillomavirus (HPV) [16,17]) and myriad other virally induced cancers [18–20].

In the broadest sense, a pathogen can be defined as any substance capable of causing disease [21]. Under this definition, pathogens need not be replicative, and could include toxins, food allergens, and dietary antigens responsible for chronic inflammation, such as gluten peptides in the context of celiac sprue.

Celiac sprue is a chronic enteropathy caused by dietary gluten from common food grains such as wheat, rye, and barley [22]. In sharp contrast with virtually all other dietary proteins, gluten proteins are minimally digested by the normal complement of gastrointestinal proteases, yielding proteolytically resistant peptides that accumulate in the proximal small intestine upon gastric emptying of a gluten-containing meal [23,24]. An inflammatory response to these metastable peptides is triggered in genetically susceptible individuals that is initially localized to the small intestine but that eventually leads to a systemic humoral response against gluten [25]. Although the clinical signs and symptoms of celiac sprue are highly variable, in the small intestine this inflammatory response causes flattening of the villi, crypt hyperplasia, and intraepithelial lymphocytosis, which in turn leads to nutrient malabsorption and/or chronic diarrhea [26,27]. If undiagnosed and untreated, this chronic inflammation is associated with the increased incidence of T cell lymphoma of the small intestine [28,29]. In most celiac patients, adherence to a gluten-free diet reverses damage to intestinal structure and function, while reintroduction of dietary gluten results in relapse [30].

In this review, we describe the unique attributes of immunotoxic gluten peptides that enable them to enact disease in celiac sprue patients. Interestingly, many parallels can be drawn between these attributes and those of more classical (infectious) pathogens. Our intent in making such a comparison is not to advocate reclassification of gluten peptides as pathogens. Rather, we hope to promote a dialogue across scientific communities that leads to a deeper understanding of celiac pathogenesis as well as to a keener recognition of salient characteristics of established and emerging pathogens.

## Gluten Peptides as Non-Replicative Pathogens

To cause disease in a susceptible host, infectious pathogens must encounter that host (exposure), overcome barriers to infectivity, access a privileged niche, colonize, and ultimately cause damage to the host either directly, through toxin secretion, or indirectly, through activation of a self-injurious host immune response. In many cases, additional steps, such as activation of the infectious pathogen to a more virulent form and subversion of host processes toward a virulent end, are prerequisite to disease as well.

**Figure 1:**
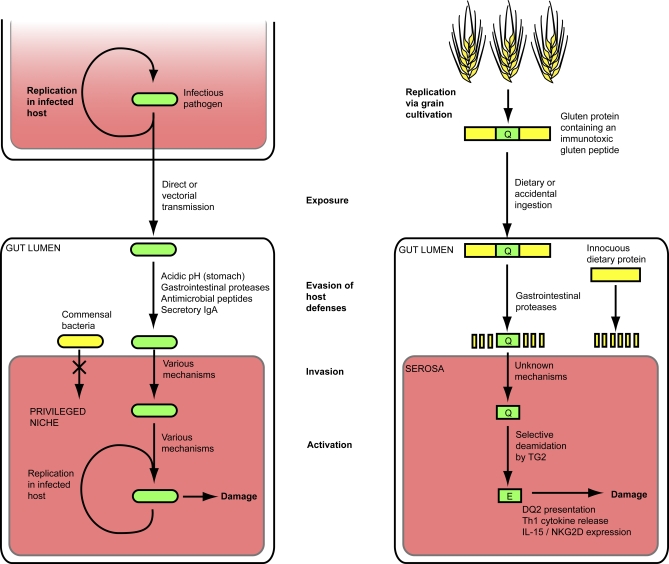
Generalized Schematic Depiction of the Parallels between Infectious Pathogen Transmission to a Susceptible Host via the Gut (Left Panel) and Celiac Sprue Pathogenesis in a Susceptible Individual (Right Panel) Infectious pathogens (green rounded rectangle; left panel) replicate within a privileged niche in an infected individual, and are then transmitted, either directly or via a pathogen-bearing vector, to another susceptible host (white box; left panel). To infect this new host, the pathogen must evade host defenses, invade across host barriers into a privileged niche (pink box), and in some cases become activated to a virulent form. Damage is caused to the host by pathogen- and/or host-mediated processes, while replication within the infected host enables further transmission. Commensal microbes (yellow rounded rectangle) cannot access privileged niches and do not cause disease. Similarly, immunotoxic gluten peptides, clustered in proline/glutamine-rich regions of gluten proteins (protein depicted as yellow rectangle containing immunotoxic peptide in green, right panel), cause celiac sprue in susceptible individuals (white box; right panel) by evading gastrointestinal proteolysis, invading across the intestinal epithelium by unknown mechanisms, and, in some peptides, becoming activated by TG2 (represented by Q (glutamine) → E (glutamate) modification), resulting in a deleterious immune response. Most dietary proteins (yellow rectangle) are proteolyzed by gastrointestinal proteases and do not enact pathogenic effects. In contrast to infectious pathogens, gluten peptides have no replicative capacity within afflicted individuals. Instead, these immunotoxic peptides are propagated by grain cultivation and transmitted to celiac sprue patients via intentional or accidental ingestion in the course of their diet.

The gluten-induced pathogenesis of celiac sprue proceeds through a remarkably similar trajectory (Figure 1). Gluten peptides enter the body as components of common dietary grains, evade destruction by gastrointestinal proteases, invade across the intestinal epithelium intact, become activated to a more immunotoxic form via enzymatic deamidation, and exert both innate and immunogenic effects in susceptible individuals, leading to disease. At two stages in this process, the immunotoxicity of gluten peptides is increased through the actions of endogenous enzymes. Gluten peptides can thus be thought of as non-replicative pathogens, bearing many similarities to infectious pathogens, with the exception of their inability to replicate or colonize an afflicted individual.

### 

#### Exposure and susceptibility to the pathogen.

The first step in any pathogen-initiated disease is exposure of the host to the pathogen. Whether this exposure results in disease depends both on the virulence of the pathogen and on the susceptibility of the host [4]. Highly virulent infectious agents, such as human immunodeficiency virus (HIV), cause disease in virtually all exposed individuals, such that the primary determinant of disease incidence is the frequency of new exposures. However, exposure to most pathogens is necessary but not sufficient to cause disease, and the genetic and conditional susceptibility of the host are additional determinants of disease progression [31]. Indeed, less virulent pathogens may be in frequent contact with potential hosts but cause symptomatic disease in only a small fraction of those exposed. For example, persistent H. pylori infection is present in roughly half of the world's population, and in up to 80% of populations in developing areas, yet only 10%–20% of those infected experience peptic ulcer disease, and only 1% develop gastric cancers [32]. As a corollary to the necessity of pathogen exposure toward infectious disease, the eradication or clearance of the pathogen from the host results in attenuation of disease symptoms.

Gluten peptides are similar to H. pylori and other high prevalence, low virulence pathogens in that they are ubiquitous but cause disease only in susceptible individuals. Gluten-containing grains such as wheat, rye, and barley are extremely common dietary components in modern agricultural societies. Additionally, many ostensibly gluten-free products contain gluten contaminants due to the use of these proteins in food processing, as well as in certain non-food items such as cosmetics and household cleaning products. Despite the nearly universal presence of gluten as a dietary protein source, the prevalence of celiac sprue is established by serological screening to be 1:100–1:200, and many of these cases are asymptomatic and undiagnosed [33].

The human class II major histocompatibility complex (MHC) plays a prominent role in determining genetic susceptibility to disease. Human leukocyte antigen (HLA) DQ2 is associated with over 90% of diagnosed celiac sprue patients, while HLA DQ8 is present in virtually all other cases [34]. Nevertheless, the HLA region confers only 40% of the genetic risk for celiac sprue, suggesting that other inherited susceptibility factors remain to be identified [34]. Moreover, the onset of symptomatic disease temporally varies between childhood and late adulthood among diagnosed patients, suggesting that gluten alone may not be sufficient to trigger the initial onset of disease. Other environmental factors that may confer conditional susceptibility on afflicted individuals include gastrointestinal surgery, pregnancy, and innate immune system activation caused by microbial colonization of the proximal small intestine [35,36]. Once symptoms of celiac sprue manifest, however, gluten is sufficient to reinitiate and sustain the disease thereafter. The only currently available treatment for celiac sprue is a lifelong gluten-free diet. While difficult to maintain due to the reasons stated above, dietary exclusion of gluten causes symptomatic remission in most celiac patients [37].

#### Evasion of “host” defenses.

Due to their route of entry, gluten peptides are most readily compared to pathogens of the gastrointestinal tract. Such pathogens encounter an extremely hostile environment that destroys any exogenous agents not uniquely suited to survive. In the stomach, gastric juices containing a mixture of hydrochloric acid, lysozyme, and pepsin prevent infection by ingested bacteria [38], and may attenuate the infectivity of low doses of prions [39]. Gastrointestinal microbes have devised a variety of strategies to surmount these defenses. H. pylori colonizes the gastric mucosa by producing a urease that hydrolyzes gastric urea to ammonia and carbon dioxide, thereby buffering its periplasmic pH, as well as that of its surroundings [40,41]. Yersinia enterocolitica employs a similar tactic en route to its site of colonization in the intestine [42], while Shigella flexneri and Escherichia coli are resistant to pH values as low as 2.0–2.5, and Salmonella typhimurium undergoes an acid tolerance response to endure transient acidity [43].

Gastrointestinal proteases are the primary defense against potentially toxic dietary proteins. Gastric pepsin, pancreatic proteases trypsin, chymotrypsin, elastase, and carboxypeptidase, as well as exopeptidases anchored to the mucosal surface, cooperatively and rapidly digest most dietary proteins into single amino acids, di-, and tri-peptides [44,45]. These digestion products are too small to elicit an immune response, and are absorbed across the mucosa for their nutritional value. By contrast, gluten proteins are incompletely digested by gastrointestinal proteases [23,24,46]. The structural basis for this proteolytic resistance has been elucidated. Gluten proteins are unusually rich in proline (∼15%) and glutamine (∼35%) residues, particularly in those regions identified as immunotoxic in celiac sprue [47]. Cleavage adjacent to proline is highly disfavored for most proteases, and glutamine is not a preferred residue for any of the endoproteases found in the gut. Consequently, peptides of sufficient length to precipitate an immune response evade gastrointestinal digestion to reach the intestinal epithelium unscathed (e.g., a 33-residue peptide [24]).

The proteolytic resistance of gluten proteins may be further enhanced by their assembly into insoluble aggregates, a property imparted by their primary sequence. Wheat gluten comprises two protein groups, the monomeric gliadins, and the low and high molecular weight (LMW and HMW) glutenins [47]. Homologs with similar properties exist in barley and rye [47]. Both gliadins and glutenins contain intrachain disulfide bonds and exhibit poor aqueous solubility, both of which are likely to reduce their proteolytic susceptibility in the gut. In contrast to gliadins, however, glutenins are also extensively cross-linked by interchain disulfide bonds, resulting in the formation of 500 kDa to 10 MDa aggregated protein complexes [48]. These huge gluten networks are further stabilized by hydrogen bonding between the glutamine-rich hexapeptide and nonapeptide repeats that compose ∼80% of each ∼100 kDa HMW glutenin subunit (Table 1) [49]. Glutamine-rich repeats, such as those present in gluten, are predictive of aggregation propensity, and have been used to identify novel prion-forming proteins [50]. Indeed, repetitive sequences identified in gluten bear remarkable similarity to those present in two extensively studied prions, Sup35 from yeast and mammalian PrP (Table 1) [51,52]. In Sup35, these repeats are necessary [53] and sufficient [54] for prion propagation, whereas PrP octarepeats do not appear to be essential for prion-related pathology [55]. Nonetheless, PrP octarepeats can functionally replace Sup35 repeats to promote protein aggregation in yeast [56], and transgenic PrP proteins lacking the octarepeats exhibit significantly reduced conversion to their pathogenic conformation, PrP^Sc^ [55]. The PrP^Sc^ form is characterized by increased β-sheet content, reduced solubility leading to aggregation, and increased proteolytic resistance with respect to the properly folded form [57,58]. Thus, glutamine-rich oligopeptide repeats contribute to prion aggregation, which in turn imparts partial proteolytic resistance on these aberrant proteins. While the majority of immunotoxic gluten epitopes identified to date derive from monomeric gliadins, immunotoxic sequences are also present in glutenins [59–63]. Accordingly, the aggregation of these proteins is likely to contribute to disease by protectively shuttling immunotoxic epitopes through the alimentary tract until their eventual release.

**Table 1:**
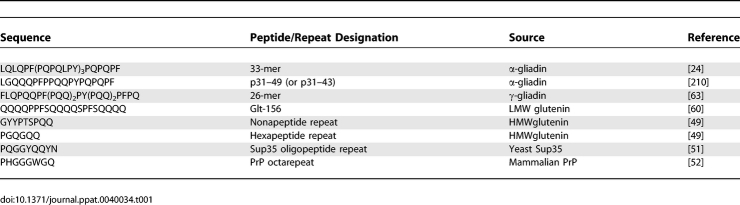
Selected Proline/Glutamine-Rich Repetitive Gluten Sequences Compared to Prion Oligopeptide Repeats

Interestingly, it is via the action of endogenous gastrointestinal proteases that immunotoxic peptides are released to enact their harmful effects. Insofar as the intact dietary gluten proteins harboring these peptides would be less efficiently transported across the intestinal epithelium to be presented to the immune system than their immunotoxic fragments, it can be said that gastrointestinal proteases facilitate the pathogenesis of celiac sprue. This subversion of the normal process of nutrient digestion toward a pathogenic end bears resemblance to the tactic of host subversion commonly employed by infectious pathogens. A relevant example of this is the trypsin-catalyzed cleavage of rotavirus protein VP4 into fragments VP5 and VP8 [64], the latter of which disrupts the barrier function of the epithelium, facilitating viral entry [65]. A second instance in which gluten peptides employ endogenous proteins to augment their pathogenicity will be discussed later in this review.

#### Invasion across intestinal epithelium.

Pathogens that survive the harsh conditions in the stomach and proximal gut must overcome yet another barrier to pathogenicity in the form of the intestinal epithelium. The epithelial layer of the small intestine is a villous structure in which polarized enterocytes are joined together at their apical surface by transmembrane protein complexes called tight junctions and adherens junctions [66,67]. Tight junctions are located most apically and are composed of junctional adhesion molecules (JAM), claudins and occludin, while adherens junctions lie just below the tight junctions and are formed by homotypic interactions of E-cadherin. The tight and adherens junctions are connected to the actin cytoskeleton by associated proteins zonula occludins 1 (ZO-1) and α/β-catenins, respectively. These junctional complexes allow diffusion of small molecules between contiguous cells via the paracellular pathway, while preventing the entry of microbes and potentially antigenic macromolecules. Absorption across enterocytes, via the transcellular pathway, is likewise selective toward dietary protein-derived amino acids, di-, and tri-peptides, which are taken up by specific transporters on the apical membrane [44,45]. Larger proteins and microbes are deterred from being transported via this pathway by secretory immunoglobulin (Ig)A and by the mucus layer coating the apical membranes of these cells. The mucosal epithelium is thus selectively permeable to nutrients while acting as a barrier to pathogens and potentially antigenic macromolecules.

The ability to invade across the epithelium to access a privileged niche is a key determinant of whether a gastrointestinal microbe is pathogenic or commensal [2]. Transepithelial invasion by microbes commonly involves disruption of the apical-junctional complex [68,69]. For example, Vibrio cholerae [70] and Bacteroides fragilis [71] secrete proteases that cleave the extracellular domains of occludin and E-cadherin, respectively. Adenovirus and rotavirus, among many other viral pathogens, also directly target junctional proteins, disturbing their proper function [65,68]. A more indirect approach is taken by H. pylori, which increases paracellular permeability in gastric epithelia by translocating the effector protein CagA into the epithelial cell to which it is adhered. Once inside, CagA recruits ZO-1 and JAM to the site of bacterial attachment, interfering with the assembly of functional tight junctions [72]. Enteropathogenic *E. coli* similarly disrupts tight junctions between enterocytes using the effector proteins Map and EspF [73]. Still other pathogenic microbes that cannot overcome the apical junctional complex bypass it altogether by availing themselves of preexisting points of entry. For instance, Listeria monocytogenes exploits the transient luminal exposure of its host receptor, E-cadherin, at sites of epithelial cell extrusion at the tips of intestinal villi [74,75]. Shigella flexneri, Salmonella typhimurium, and Yersinia pseudotuberculosis all cross the epithelium by being captured by specialized antigen-sampling epithelial cells, called M cells, and subsequently escaping macrophage-mediated destruction once translocated [76]. Infectious prions are also observed to invade across enterocyte layers [77], and may do so using M cells as a portal [78].

The pathways and mechanisms by which gluten peptides are transported across the intestinal epithelium are not yet known. During active disease (i.e., on a gluten-containing diet), the architecture of the celiac epithelium is grossly perturbed. Intestinal biopsies of lesions exhibit villous flattening and crypt hyperplasia as well as increased enterocyte apoptosis, suggesting the integrity of the epithelium may be compromised [79,80]. Moreover, the jejunal tight junction structure is morphologically altered in celiac sprue patients [81], and molecular analysis of these junctions has recently revealed that both occludin and E-cadherin fail to localize properly [82]. As a result, untreated celiac patients exhibit increased permeability toward small molecules and sugars used as paracellular markers [83–87]. Transcellular transport is also upregulated during active enteropathy, as evidenced by the increased endocytic uptake of gluten peptides across the apical membrane of celiac jejunal biopsy enterocytes [88]. Concomitant with this increased uptake, the apical-to-basolateral transcellular flux of specific gluten peptides and their antigenic metabolites across untreated celiac patient jejunal biopsies is also increased relative to controls [89,90].

The increase in both paracellular and transcellular permeability observed during active celiac sprue is due, at least in part, to the Th1 cytokines interferon-γ (IFN-γ) and tumor necrosis factor α (TNF-α). These proinflammatory cytokines are upregulated in active celiac lesions [91–93] as well as in other inflammatory bowel diseases in which the epithelial barrier is disrupted [94]. Their effects on epithelial monolayer permeability have been extensively studied *in vitro* [95]. In cultured T84 epithelial monolayers, IFN-γ causes actin cytoskeletal rearrangement and tight junction protein internalization [96,97], resulting in increased paracellular permeability [98,99]. TNF-α also disrupts tight junction assembly [100], and potentiates the permeabilizing effects of IFN-γ [99]. Additionally, IFN-γ increases the transcellular flux of intact proteins across HT29-19A epithelial cell monolayers [101]. Taken together, these results show that IFN-γ and TNF-α induce the same epithelial alterations that are characteristic of celiac lesions, in which these cytokines are overexpressed, and are therefore at least partially responsible for the increased permeability of the gut during active inflammation.

However, these results leave open the pathways and mechanisms by which gluten peptides first cross the intestinal epithelium to come into contact with the underlying lymphoid tissue and thereby initiate inflammation. This event may depend on genetic predisposition toward impaired gut barrier function, environmental factors that prime the intestine for uptake of gluten, or preexisting routes of luminal antigen uptake that are shared between celiac patients and healthy individuals. Of course, a combination of these factors may be at play.

To date, there is minimal evidence for celiac patients possessing genetic defects in gut barrier function. Defects in epithelial tight junction structure [81] and paracellular permeability [83–85] persist after treatment with a gluten-free diet, as does the increased transcellular uptake of gliadin into enterocytes [102]. However, due to the difficulty of ensuring a completely gluten-free diet in human patients, it is not clear whether these persistent defects reflect genetically encoded traits, incomplete recovery of the gut, or a continued inflammatory reaction to low levels of dietary gluten [103–106]. To circumvent this issue, longitudinal studies examining permeability in potentially gluten-sensitive individuals prior to dietary intake of gluten and the onset of inflammation are needed. Due to the practical limitations of conducting such studies in humans, the investigation of this question awaits an animal model for celiac sprue, in which gluten intake can be strictly controlled. Of course, a genetically tractable animal model, such as a mouse, will allow for a more sophisticated toolbox to be directed at this question.

Alternatively, there may not be any genetic determinants of celiac sprue related to the transepithelial transport of gluten peptides. Instead, other environmental factors, or gluten itself, may contribute to disease onset by attenuating the barrier function of the intestine. Gastrointestinal infections can permeabilize the gut by causing inflammation or by other mechanisms. For example, in a cell culture model of H. pylori infection, the transcellular flux of intact protein is increased due to urease-dependent impairment of lysosomal protein degradation [107]. Physiologically relevant temperature increases, such as may occur in the context of a fever or bacterial infection, may also permeabilize epithelial monolayers by increasing paracellular flux [108], thereby rendering the intestine conditionally susceptible to opportunistic invasion by gluten peptides. Interestingly, certain gluten peptides may even have an intrinsic ability to directly affect epithelial permeability. Pepsin-trypsin (PT)-gliadin digests induce production of TNF-α in cultured monocytic cell lines [109]. Moreover, apically administered PT-gliadin causes actin cytoskeletal rearrangement, changes in expression and localization of tight junction proteins, and increased permeability toward paracellular markers in cultured epithelial monolayers [110,111].

Finally, there exist multiple pathways by which small amounts of dietary proteins are regularly transported intact across the healthy intestinal epithelium [112,113]. Various orally administered proteins are observed to cross the epithelium in healthy individuals while retaining their immunoreactivity [114,115] and biological activity [116]. Gluten is among these, as anti-gliadin antibody-reactive proteins can be detected in the breast milk and sera of healthy human mothers on a gluten-containing diet [117]. This low-level intact transport likely operates through a non-degradative transcellular pathway, either through M cells, or following non-specific endocytic uptake at the apical enterocyte membrane [113]. Larger gluten peptides, such as the 33-mer, may additionally be transported through enterocytes via the lysosomal pathway, delivering antigenic fragments to the serosa [89,90]. Receptor-mediated mechanisms for gluten peptide transport have also been proposed, and several proteins implicated in the pathology of celiac sprue have been suggested as candidate receptors. These include gluten-specific IgA and MHC class II molecules [88], as well as transglutaminase 2 (TG2) [118]. The existence of IgA-deficient individuals with celiac sprue suggests that anti-gliadin IgA is not an essential factor for this endocytic gluten uptake [119]. The MHC class II products HLA DR and DP are upregulated in the apical epithelium during active celiac sprue, but expression of the disease-associated HLA DQ products is restricted to the lamina propria [120]. Dendritic cells present in the lamina propria express both surface TG2 and HLA DQ molecules [121], and can extend dendrites through the epithelial layer to directly sample luminal antigens [122]. The identification of a subset of mucosal dendritic cells that can activate gluten-reactive T cells raises the intriguing possibility that gluten peptides may invade across the intestinal epithelium via the same cells that present them to the immune system [118].

##### Activation to pathogenic form.

There are numerous examples of pathogens that do not achieve full virulence until being activated to a more pathogenic form. The most striking example among these may be prions. In their correctly folded form, called PrP^C^, prions are endogenous membrane proteins that are entirely innocuous. Once conformationally activated, either by spontaneous misfolding or by the catalytic action of the misfolded form, PrP^Sc^, these infectious proteins are responsible for a variety of neurodegenerative diseases [10]. Other examples of pathogenic activation include the integration of HPV type 18 prior to the development of high-grade cervical intraepithelial neoplasia [16], viral protease-mediated cleavage of *gag* and *gag-pol* precursor polyproteins as a prerequisite to the maturation of infectious HIV particles [123], and the conferral of virulence on formerly commensal bacteria via horizontal transfer of pathogenicity islands [124].

Gluten peptides must be deamidated at select glutamine residues before they achieve full immunotoxicity in the context of celiac sprue [125]. Although it was initially thought that this deamidation occurred due to the acidic pH in the stomach, it has since become clear that gliadin peptides are selectively modified by the endogenous enzyme, TG2 [126,127]. TG2 is a pleiotropic enzyme found both intracellularly and extracellularly in many tissues and organs, including the small intestine, where it is upregulated during active celiac sprue [128–130]. In a Ca^2+^-dependent manner, TG2 catalyzes the transamidation of specific glutamine carboxamide sidechains with amine donors, such as the ɛ-amino group of lysine, forming isopeptide bond crosslinks between proteins. When water replaces the amine donor as the nucleophile, TG2 instead deamidates these glutamines to glutamates, introducing a negative charge at each modified position [131]. It is this latter activity that enables TG2 to activate gluten peptide immunoreactivity. The same proline/glutamine-rich sequences that render gluten peptides resistant to gastrointestinal proteolysis also make them excellent substrates for TG2 [132,133]. Following TG2-mediated deamidation at select Gln residues, these peptides bind with increased affinity to disease-associated HLA DQ2 molecules [134,135], and thereby possess increased stimulatory capacity toward DQ2-restricted gluten-reactive T cells [136,137]. As will be discussed shortly, much of the damage that occurs in celiac sprue is mediated by this disease-specific T cell response. Thus, the deamidation of immunotoxic gluten peptides by endogenous TG2 constitutes the second point at which normal cellular processes are subverted toward a pathogenic end in celiac sprue.

Notably, the transamidase activity of TG2 is also implicated in celiac sprue. During active disease, celiac patients have circulating antibodies not only against gluten epitopes, but also against TG2 [138]. Since TG2 forms covalent complexes with gluten peptides [139], it has been proposed that intestinal gluten-reactive T cells can provide co-stimulation to B cells expressing TG2-specific antibodies as part of an autoimmune humoral response [140]. Upon treatment with a gluten-free diet, anti-TG2 autoantibody levels decline. Whether anti-TG2 autoantibodies play a role in disease pathogenesis or are simply bystanders is not yet clear. However, these highly disease-specific antibodies do serve an important role in serological screening for celiac sprue [26].

Although TG2 is found in the lamina propria and the brush border of enterocytes, the precise location and context in which TG2 encounters and deamidates gluten peptides is not yet known.

#### Initiation of deleterious immune response.

The ultimate defining characteristic of pathogens is that they contribute to disease. It has been suggested that pathogens can be classified according to the damage their presence inflicts on a host relative to the strength of the host's immune response [4]. Those microorganisms classically termed opportunistic cause disease only in the context of compromised immunity. Diseases caused by toxin-producing pathogens comprise damage mediated both by the pathogen and by the host's immune response, the contributions of each depending on the potency of the toxins produced, as well as on the pathogen's ability to avoid provoking a strong immune response. At the far end of this continuum, pathogens that produce no toxins of their own precipitate disease in the context of a strong, host-damaging inflammatory response. Gluten peptides are examples of this last category.

Celiac sprue is a chronic inflammatory disease. In infectious disease, chronic inflammation occurs when a pathogen continually evades an active immune response, for instance by resisting phagocytic engulfment or by aggressin-mediated killing of macrophages. This inflammation persists, resulting in significant tissue damage, until the colonizing pathogen is cleared. As non-replicative immunotoxins, ingested gluten peptides possess no capacity to colonize the gut. Instead, chronic inflammation persists in the celiac gut due to the continual dietary reintroduction of immunotoxic peptides from an exogenously replicating pool of cultivated gluten-containing grains. With adherence to a gluten-free diet, the immunotoxin is cleared, and inflammation resolves.

**Figure 2:**
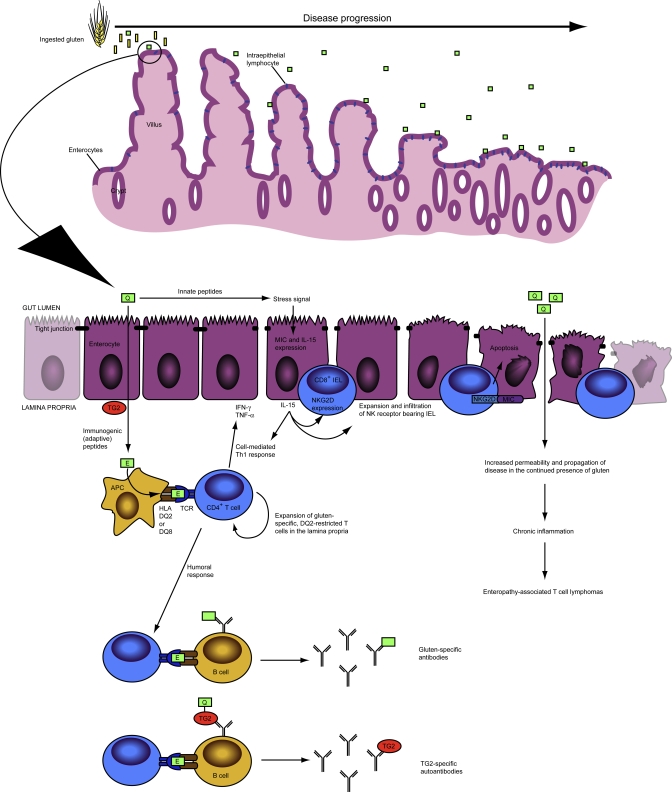
Adaptive and Innate Immune Responses to Gluten in Celiac Sprue Ingested gluten is digested to innocuous amino acids (yellow boxes) and proteolytically resistant, immunotoxic gluten peptides (green) in the small intestine. Immunogenic gluten peptides access the lamina propria by unknown mechanisms and are deamidated by TG2. These deamidated peptides are loaded onto HLA DQ2 (or DQ8) and presented on the surface of antigen-presenting cells (APC) to gluten-specific, DQ2-restricted CD4^+^ T cells in the lamina propria, causing their activation and clonal expansion. Activated T cells mediate the humoral response, by giving help to both gluten-specific and TG2-specific B cells, as well as the cell-mediated Th1 response, which, through the secretion of proinflammatory cytokines such as IFN-γ and TNF-α, disrupts tight junction integrity. In parallel, innate peptides act through unknown mechanisms as a stress signal toward enterocytes, inducing expression of MIC and IL-15. IL-15 promotes the infiltration of CD8^+^ IEL into the epithelium, and arms them with the NK receptor NKG2D. IL-15 may also influence the Th1 response. Intraepithelial lymphocytes bearing NKG2D target MIC-expressing enterocytes for killing via apoptosis, causing destruction of the epithelial layer, and villous flattening. The combination of enterocyte apoptosis and tight junction disruption renders the epithelium more permeable, thereby facilitating access of gluten and propagation of the disease. In the continued presence of dietary gluten, chronic inflammation persists, and, in a small percentage of patients, results in enteropathy-associated T cell lymphomas. TCR, T cell receptor.

The mechanisms by which gluten peptides precipitate inflammation in the celiac gut are only recently becoming clear. Over the past decade, we have begun to appreciate that celiac pathogenesis involves a complex interplay between adaptive and innate responses, each of which is mediated by a distinct class of immunotoxic gluten peptides (Figure 2) [141–143].

The first of these classes provokes the T cell–mediated adaptive response. These immunogenic peptides, typified by the 33-mer (Table 1) [24], are excellent substrates for TG2, and, once deamidated, are potent activators of gluten-specific, DQ2-restricted CD4^+^ T cells in the lamina propria [25]. Activated CD4^+^ T cells enact a Th1 response, secreting IFN-γ and other proinflammatory cytokines, as well as give help to the B cell–mediated humoral response against both gluten and TG2. Due to the remarkable concordance between the role that TG2 plays in increasing these immunogenic peptides' affinity for DQ2, the identity of TG2 as the target of the autoantibody response, and the strong genetic association of DQ2 with disease, research into celiac pathogenesis has largely focused on the adaptive branch of the immune response. However, the gluten-specific adaptive immune response is thought to be insufficient on its own to explain why CD4^+^ lamina propria T cells trigger an inflammatory Th1 response [91–93]. It also does not provide an explanation for the characteristic expansion of intraepithelial lymphocytes (IEL), the majority of which are CD8^+^, seen in active celiac intestinal epithelium. Finally, gluten-specific adaptive immunity cannot account for how enterocytes lining the gut are targeted for destruction during active disease.

These outcomes may be explained by the involvement of a non-T cell–mediated innate response, induced by a second class of immunotoxic gluten peptides. The best characterized of these innate peptides, p31–43 (or p31–49) (Table 1), is distinguished from immunogenic gluten peptides in that it does not stimulate gluten-reactive CD4^+^ T cells [127,144]. Instead, this peptide acts directly on epithelial cells as a stress signal, causing increased enterocyte expression of both interleukin-15 (IL-15) and the non-classical MHC class I molecules, MIC and HLA-E, when intestinal biopsies derived from treated celiac patients are exposed to it [144,145]. IL-15 promotes IEL expansion [146], and induces the expression of natural killer (NK) receptors NKG2D and CD94 on the surface of effector IEL [147,148]. These NK receptor-bearing IEL are targeted to kill epithelial cells via NK receptor engagement of MIC stress markers on the surface of enterocytes [145,149]. The *in vivo* relevance of these effects is underscored by the presence of upregulated IL-15 [146,150,151], increased MIC expression on enterocytes [145,149], and CD94^+^ IEL infiltration [147] in the intestinal epithelium of active celiac patients. Thus, innate gluten peptides cause damage to the gut by inducing epithelial stress and IL-15 expression, which in turn lead to IEL infiltration and targeted killing of MIC-expressing enterocytes by NK receptor^+^ IEL in a manner independent of T cell receptor specificity. The mechanism by which these peptides induce stress in epithelial cells is still not known. However, inactive TG2 on the surface of enterocytes may mediate this effect, since neutralization of surface TG2 with the monoclonal antibody 6B9 attenuates the innate effects of p31–43 [152].

Innate immunity may also play a role in directing the gluten-specific adaptive response toward a Th1 cytokine profile. Since IL-12, a major promoter of Th1 differentiation, is absent in celiac sprue [92], other cytokines must mediate this Th1 differentiation. Two possible candidates are IFN-α and IL-15. Increased levels of IFN-α are present in active celiac mucosa relative to controls [153], and the onset of celiac symptoms during treatment with this cytokine has been reported [153,154]. Additionally, IFN-α upregulates the production of proinflammatory cytokines IFN-γ and TNF-α by activated intestinal T cells, and causes hyperproliferation of crypt cells in intestinal biopsies [155]. Whether IFN-α is induced by exposure to p31–43 is not yet known. In *ex vivo* biopsy culture experiments, IL-15 is known to be induced by p31–43, and it also drives secretion of IFN-γ and TNF-α by IEL [146]. Moreover, p31–43 potentiates the activation of lamina propria T cells by immunogenic peptides, and this effect is mitigated by IL-15 inhibition [144], suggesting both p31–43 and IL-15 influence the course of the gluten-specific adaptive immune response.

Cancer is a well-established complication of chronic inflammation in response to the persistent presence of a pathogen [156,157]. It is therefore not surprising that untreated celiac sprue patients have an elevated risk for developing rare non-Hodgkin lymphomas (odds ratio = 3.1) [29], particularly enteropathy-associated T cell lymphoma (EATL), while exclusion of dietary gluten reduces this risk [28]. A small proportion of celiac sprue patients are refractory to treatment with a gluten-free diet, and 75% of these patients exhibit a clonally expanded population of abnormal IEL, resembling lymphomas present in EATL [158]. Thus, refractory sprue may represent an early stage of EATL, wherein clonally expanded, cytotoxic IEL continue to cause intestinal damage in the absence of dietary gluten. Although it has been shown that IL-15 preferentially promotes the clonal expansion and survival of these abnormal IEL [151], it is not yet clear what other factors contribute to the progression of refractory sprue and EATL.

## Animal Models for Celiac Sprue

In the field of pathogen research, microbiologists have repeatedly shown that a test tube is a highly predictive model for infectious disease. For example, the discovery of many antibiotics, including penicillin and streptomycin, has relied on observations of their *in vitro* effects on cultured bacteria. Likewise, pathogenic infection models in mammalian cell culture have facilitated numerous insights into pathogen motility, attachment, invasion, and production of virulence factors. In an analogous fashion, a combination of biochemical and cell culture assays has elucidated many attributes of gluten peptides that enable their immunotoxicity. These include *in vitro* models for gluten peptide gastrointestinal digestion [23,24,159,160], transepithelial transport [89], TG2-mediated deamidation [126], HLA-DQ2 binding and presentation [134,135,161], T cell activation [136,162], and enactment of innate immune responses through direct toxic effects [144,145].

However, many inquiries in pathogen research, including studies on transmission, vaccination, drug bioavailability, and pathogen-mediated damage at the level of whole organs and tissues, are critically dependent on animal models exhibiting specific characteristics of corresponding human diseases. A complete understanding of celiac sprue will be similarly dependent on *in vivo* models that recapitulate specific aspects of the human disease's complex etiology. Such animal models may be of particular importance in identifying non-HLA genes contributing to disease susceptibility, in elucidating mechanisms of peptide transport and immune-mediated intestinal damage, and in evaluating proposed therapeutics on the basis of how well they attenuate clinical, histological, and serological readouts of disease.

While a bona fide animal model for celiac sprue is still lacking, both natural and engineered reactions to dietary gluten that mimic certain aspects of the human disease have been reported in laboratory rabbits, Irish setter dogs, non-human primates, and transgenic mice (Table 2). A majority of laboratory rabbits fed a gluten-containing diet produce anti-gliadin IgG, in contrast to wild hares, which do not eat gluten [163] (M. Bethune, unpublished results). However, these rabbits do not produce anti-gliadin IgA (M. Bethune, unpublished results), and are apparently asymptomatic, suggesting that while gluten may encounter the immune system, it is not pathogenic in these animals. Gluten-sensitive Irish setter dogs are the best-characterized natural animal model, featuring both gluten-dependent diarrhea and histological lesions [164–166]. When raised on a gluten-free diet, affected animals exhibit increased gut permeability toward ^51^Cr-EDTA relative to controls. Importantly, this condition precedes overt enteropathy, suggesting the existence of a primary defect in gut permeability [166,167]. However, gluten-sensitive Irish setters do not raise antibodies against gluten even in an active state of disease, so it is not clear that this finding can be extrapolated to gluten peptides of sufficient size to be immunotoxic [168]. Moreover, the lack of MHC class II linkage with disease in these animals disqualifies them as complete models for celiac sprue [169,170]. Published observations of celiac sprue–like enteropathy in non-human primates are limited to two case reports, one in a single rhesus macaque necropsy [171], and another in a single cynomolgus monkey, the latter of which improved on a gluten-free diet [172]. More recently, a condition of gluten sensitivity has been identified and characterized in juvenile rhesus macaques (M. Bethune, J. Borda, E. Ribka, M. Liu, K. Phillippi-Falkenstein, et al., unpublished data). At a significant frequency, these animals exhibit clinical, histological, and serological signs of gluten sensitivity that resolve upon treatment with a gluten-free diet. Association of MHC class II alleles with this condition remains to be investigated. Finally, several transgenic mouse models have been engineered to mimic celiac sprue, most notably the NOD Ab° DQ8^+^ mouse, which expresses human DQ8 in an endogenous MHC class II-deficient (Ab°), autoimmune-prone (NOD) background [173]. Although this mouse model exhibits no gastrointestinal lesions or GI-related symptoms, it develops skin rashes with subcutaneous IgA deposits reminiscent of dermatitis herpetiformis, and may therefore be useful in the study of this dermatologic manifestation of celiac sprue.

**Table 2:**
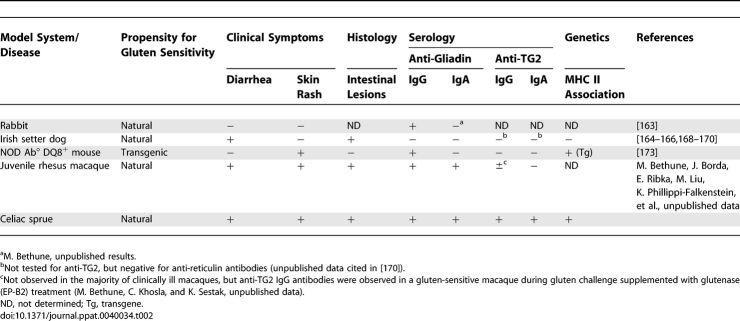
Comparison of Celiac Sprue with Proposed Animal Models for Gluten Sensitivity

## Therapeutic Intervention

**Figure 3:**
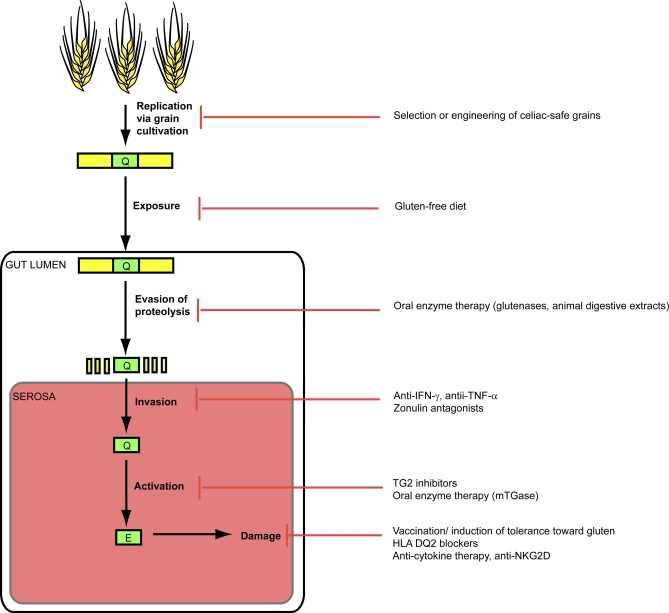
Potential Therapies Targeting Each Stage of Celiac Disease Progression Immunotoxic gluten peptides may be eliminated at the source through selection or engineering of non-toxic varieties of gluten-containing grains. Exposure to gluten peptides may be avoided by means of dietary abstention. Gluten peptides' proteolytic resistance may be countered via oral enzyme therapy with glutenases. Invasion of gluten peptides across the intestinal epithelium may be prevented by targeting mediators of epithelial permeability, such as proinflammatory cytokines IFN-γ and TNF-α. Pharmacological inhibition of TG2 represents a means of preventing the activation of native gluten peptides to their deamidated forms. Finally, the deleterious response to gluten peptides may be controlled by prophylactic vaccination, by blocking HLA DQ2-mediated presentation of gluten peptides, or by targeting the mediators of gluten-induced damage, such as IL-15 and NKG2D.

As our understanding of celiac sprue pathogenesis has increased, so too have the possibilities for therapeutic intervention in this debilitating disease. An extensive description of these emerging strategies is beyond the scope of this review, and has been provided elsewhere [174]. Instead we offer a few examples to illustrate the potential for developing therapeutics that target each stage of the pathogenic progression of gluten peptides (Figure 3).

### 

#### Exposure.

The only current treatment for celiac sprue is a life-long gluten-free diet [37]. While effective in most patients, adherence to this diet can be difficult due to its expense, the ubiquity of gluten, poor labeling of gluten-containing foods, and social constraints. Several other dietary strategies aimed at lowering exposure to the immunotoxic components of gluten have been proposed or experimentally advanced. These include using varieties of wheat with lower quantities of the immunotoxic epitopes found in the commonly cultivated Triticum aestivum variety [175], genetically engineering grains to contain less of these epitopes (proposed in [175]), or extirpating these immunotoxic epitopes during food processing with probiotics or by enzymatic means [176,177]. Proteolytic pretreatment of gluten-containing grains, analogous to pretreatment of dairy products with lactase, is not a widely employed strategy, since the primary structural components of gluten (i.e., HMW glutenins) contain immunotoxic epitopes [61,62], and the destruction of these epitopes would perturb the properties of dough. One novel strategy that may circumvent this limitation is the use of microbial transglutaminases in combination with amine blockers during food processing to prevent reactive glutamines from being deamidated by intestinal TG2 [177].

Importantly, strategies aimed at the level of exposure may offer celiac patients more options, but will not alleviate most of the difficulties associated with the gluten-free diet unless such alternative grains or grain treatments are widely instituted. In order to safeguard against unintentional gluten exposure and generally improve the quality of life for celiac patients, non-dietary strategies are needed.

#### Evasion of proteolytic digestion.

For decades, it was hypothesized that celiac patients were missing a critical peptidase, accounting for their inability to properly digest gluten. It has since been established that gluten is proteolyzed to a similar extent by celiac patients and healthy individuals, leaving certain proteolytically resistant peptides intact [24,178]. In light of this, a number of recent studies have focused on exogenous enzymes (i.e., “glutenases”) that are capable of proteolyzing ingested gluten [179,180]. Due to the high proline content of immunotoxic gluten peptides, the majority of these studies have utilized bacterial prolyl endopeptidases [181]. These enzymes have proven very effective at detoxifying gluten *in vitro* [24], but their efficacy *in vivo* may be limited by their pH profile (favoring intestinal digestion) and by their preference for shorter peptides. To improve on this approach, recent studies have explored the use of acid-active prolyl endoproteases [182,183], as well as a naturally evolved glutenase derived from barley (EP-B2) that targets sequences similar to those deamidated by TG2 [162,184]. Importantly, EP-B2 has complementary specificity to prolyl endopeptidases, and a combination therapy consisting of these enzymes was effective at destroying immunotoxic gluten epitopes *in vivo* [185]. Undefined enzyme combinations, such as are present in animal digestive extracts, also showed some benefit in a recent clinical trial, underscoring the potential for therapeutic glutenase supplementation [106].

#### Invasion.

Therapies aimed at preventing the transepithelial invasion of gluten peptides await a better understanding of the mechanisms by which gluten is transported. One potential strategy may involve the use of antibodies against proinflammatory cytokines, such as TNF-α and IFN-γ, as these have been shown to regulate the permeability of the gut. Treatment with infliximab, a monoclonal antibody directed against TNF-α, is used to similar effect in Crohn disease [186]. A key consideration is the general safety profile of such therapies, and whether it may be suitable for chronic use in the context of celiac sprue. Antagonists of another putative regulator of intestinal permeability, zonulin, have also been proposed [187]. A synthetic peptide based on this strategy increased the transepithelial resistance of intestinal mucosa in a diabetes-prone rat model [188] and is currently undergoing clinical trials in celiac sprue patients [189].

#### Activation.

The role TG2 plays in enhancing the immunotoxicity of many gluten peptides makes it a potential therapeutic target. The most obvious means of intervening at this stage of gluten pathogenicity is to inhibit TG2 activity directly, and indeed, inhibition of TG2 in cultured celiac patient–derived intestinal biopsies reduces gluten-specific T cell activation [152,190]. Pharmacological inhibitors of TG2 activity have been reviewed [191]. Recent studies have highlighted the potential for mechanism-based TG2 inhibitors to be of particular benefit in treating celiac sprue [192–194]. One of these inhibitors, KCC009, is well tolerated in rodents, when dosed chronically [193,195]. As an alternative to inhibiting TG2, it may be possible to block deamidation sites in gluten peptides *in vivo*, by dual oral administration of a microbial transglutaminase and a suitable amine blocker. Microbial transglutaminases have weak deamidation activity but have broader specificities and higher reaction rates for transamidation reactions than does TG2 [196]. Additionally, they are active at physiological temperatures over a wide range of pH values [197], and can utilize gluten as an acyl donor in transamidation reactions [198]. A key consideration for this strategy is whether microbial transglutaminases can target the same gluten epitopes that are deamidated by TG2. In this regard, it is notable that gluten pretreated with microbial transglutaminase and an amine donor prior to TG2 treatment induces less IFN-γ production by celiac patient biopsy-derived intestinal T cells relative to gluten receiving no pretreatment [177]. This suggests that the specificities of these enzymes do indeed overlap to some extent.

A major caveat to approaches focusing on preventing gluten deamidation is that some non-deamidated gluten peptides can induce T cell responses from celiac patient biopsies, suggesting that TG2 inhibition may not by itself protect celiac patients from ingested gluten [60,137].

#### Immune response.

Efforts to intervene in the deleterious immune response in celiac sprue can be focused at three levels: inoculation against the induction of a response, blocking the response *in situ*, or mitigating the effects of the response once it is initiated.

The first of these approaches has shown some early promise in a DQ8^+^ mouse gluten sensitivity model, where intranasal administration of a recombinant α-gliadin protein was shown to down-regulate lymph node T cell proliferation and IFN-γ production in response to subsequent parenteral gluten immunization [199]. Oral administration of nontoxic gluten peptide analogues may represent an additional route by which oral tolerance can be generated [200], though this has not been experimentally demonstrated. To block the immune response *in situ*, peptidomimetic inhibitors that bind HLA DQ2 but are not recognized by gluten-specific T cell receptors can be designed using the crystal structure of HLA DQ2 bound to a gluten peptide as a guide [135,201]. By building these inhibitors from a gluten peptide scaffold, it is hoped that such DQ2 blockers will possess similar proteolytic resistance, bioavailability, and affinity for DQ2 as immunotoxic gluten peptides. Indeed, a recent study has identified two prototypical high affinity DQ2 blockers that inhibit gluten peptide presentation by fixed antigen-presenting cells in T cell proliferation assays [201]. Finally, the recent expansion in our understanding of the role that innate immunity plays in mucosal damage provides us with several potential targets for attenuating the inflammatory response once it has been initiated. One of these is the NKG2D receptor expressed on the surface of intestinal CD8^+^ IEL. Antagonism of this receptor impairs the expansion and function of pancreatic autoreactive CD8^+^ T cells in the NOD mouse model of diabetes, thereby preventing disease [202]. A similar strategy has been proposed for the treatment of celiac sprue [145,149]. Another promising strategy may involve neutralizing those cytokines (or their receptors) that mediate tissue damage during inflammation, including IL-15 [144–146,149], IFN-γ [203], IFN-α [155], and TNF-α [204]. The anti-TNF-α monoclonal antibody, infliximab, is already widely used to reduce gut inflammation in Crohn disease [205,206], and case reports of its effectiveness in treating refractory sprue have been published [207,208]. More preliminarily, the specific neutralization of catalytically inactive TG2 on the surface of epithelial cells mitigates certain features of disease *ex vivo* [152], and may represent another therapeutic inroad once this enigmatic new role for TG2 in celiac sprue pathogenesis is elucidated.

## Conclusion

Infectious pathogens adapt to and cause disease in a particular host by evolving virulence traits that provide a context-specific, selective advantage to the pathogen (e.g., by enabling it to breach a specific host epithelial barrier, or by facilitating its dispersal via induction of diarrhea) [209]. By contrast, it is difficult to imagine how the ability of immunotoxic gluten peptides to resist gastrointestinal proteolysis, to exert damaging stress on epithelial cells, to be specifically deamidated by TG2, or to engage in high affinity HLA DQ2 binding affords any increase in fitness to the grains encoding these peptides. Nonetheless, gluten peptides possess all of these attributes, each of which is a de facto virulence trait essential toward the pathogenesis of celiac sprue. Moreover, these peptides persist as human pathogens uniquely due to our purposeful cultivation of the grains that produce them, and our quite intentional exposure to them by way of diet. Gluten peptides, then, are quintessentially accidental pathogens that cause disease in the most obliging of hosts.

Consequently, the eradication of celiac sprue as a human disease is achievable through widespread adoption of natural or engineered strains of wheat, rye, and barley with less toxic properties. Unless and until this occurs, non-dietary therapies are needed to safeguard celiac sprue patients trying to maintain a gluten-free diet in the midst of ubiquitous gluten. Such therapies require a solid understanding of the mechanisms by which immunotoxic gluten peptides cause disease, and of the factors that render afflicted individuals susceptible. Many questions remain to be answered before we can claim such complete knowledge. First, celiac sprue exhibits features of chronic inflammatory, genetic, autoimmune, and pathogen-induced diseases, but the respective etiological contributions of each are uncertain. It is not clear, for instance, whether anti-TG2 autoantibodies advance pathogenesis, or if they are merely bystanders in the humoral response. Genetic predisposition for disease is clearly imparted by HLA DQ2, but 60% of the genetic risk for celiac sprue remains unattributed. A genetically tractable animal model will greatly facilitate the search for these unknown disease determinants, possibly identifying genes involved in intestinal permeability and innate immunity. Still other players involved in disease progression are known, but their site of action is not. For example, it is not clear where the selective deamidation of ingested gluten peptides occurs. Likewise, the antigen-presenting cells that present DQ2-bound gluten to CD4^+^ T cells in the lamina propria remain to be definitively identified. A major outlying question concerns the provenance of the inflammatory immune response. Celiac sprue is widely regarded as a T cell–mediated inflammatory disease, but the discovery of an IL-15-mediated innate response to gluten calls into question whether adaptive immunity alone can cause disease. Importantly, both the immunogenic 33-mer and the innate p31–49 peptide induce characteristic villous flattening and increased IEL infiltration when administered alone to celiac patients [210,211]. Here again, the reconstitution of celiac sprue in a suitable animal model will be critical toward clarifying which processes are necessary and sufficient to provoke disease. Finally, the most important question in celiac sprue research is how we can apply our knowledge of its pathogenesis toward the development of an effective non-dietary treatment, and thereby improve the quality of life for patients living with the disease.

## Supporting Information

### Accession Numbers

The GenBank (http://www.ncbi.nlm.nih.gov/Genbank/) accession numbers for gluten and prion proteins tabulated in Table 1 are α2-gliadin (CAB76964), α9-gliadin (CAB76955), γ5-gliadin (CAC94871), low molecular weight glutenin, Triticum aestivum (ABI21861), high molecular weight glutenin, Triticum aestivum (ABQ14770), mammalian prion protein PrP (P01456), and translation termination factor Sup35, Saccharomyces cerevisiae (AAS64331). 

## Abbreviations:

EATL: enteropathy-associated T cell lymphoma
EP-B2: barley endoprotease B, isoform 2
HIV: human immunodeficiency virus
HLA: human leukocyte antigen
HPV: human papillomavirus
IEL: intraepithelial lymphocytes
IFN-γ: interferon-γ
Ig: immunoglobulin
IL-15: interleukin 15
JAM: junctional adhesion molecules
LMW and HMW glutenin: low and high molecular weight glutenin
MHC: major histocompatibility complex
NK: natural killer
PT-gliadin: pepsin-trypsin digested gliadin
TG2: transglutaminase 2 (or tissue transglutaminase)
TNF-α: tumor necrosis factor α
ZO-1: zonula occludens 1
